# mTOR Is Essential for the Proteotoxic Stress Response, HSF1 Activation and Heat Shock Protein Synthesis

**DOI:** 10.1371/journal.pone.0039679

**Published:** 2012-06-29

**Authors:** Shiuh-Dih Chou, Thomas Prince, Jianlin Gong, Stuart K. Calderwood

**Affiliations:** 1 Department of Radiation Oncology, Beth Israel Deaconess Medical Center, Harvard Medical School, Boston, Massachusetts, United States of America; 2 Department of Hematology and Medical Oncology, Boston University Medical Center, Boston, Massachusetts, United States of America; Boston University Medical School, United States of America

## Abstract

The target of rapamycin (TOR) is a high molecular weight protein kinase that regulates many processes in cells in response to mitogens and variations in nutrient availability. Here we have shown that mTOR in human tissue culture cells plays a key role in responses to proteotoxic stress and that reduction in mTOR levels by RNA interference leads to increase sensitivity to heat shock. This effect was accompanied by a drastic reduction in ability to synthesize heat shock proteins (HSP), including Hsp70, Hsp90 and Hsp110. As HSP transcription is regulated by heat shock transcription factor 1 (HSF1), we examined whether mTOR could directly phosphorylate this factor. Indeed, we determined that mTOR could directly phosphorylate HSF1 on serine 326, a key residue in transcriptional activation. HSF1 was phosphorylated on S326 immediately after heat shock and was triggered by other cell stressors including proteasome inhibitors and sodium arsenite. Null mutation of S326 to alanine led to loss of ability to activate an HSF1-regulated promoter-reporter construct, indicating a direct role for mTOR and S326 in transcriptional regulation of HSP genes during stress. As mTOR is known to exist in at least two intracellular complexes, mTORC1 and mTOR2 we examined which complex might interact with HSF1. Indeed mTORC1 inhibitor rapamycin prevented HSF1-S326 phosphorylation, suggesting that this complex is involved in HSF1 regulation in stress. Our experiments therefore suggest a key role for mTORC1 in transcriptional responses to proteotoxic stress.

## Introduction

The heat shock response is a cellular reaction to proteotoxic stress that permits cell survival and repair of protein damage [1]. This response involves a number of complementary and competing interactions. Protein stress may lead to transcriptional induction of molecular chaperones known as heat shock proteins (HSPs), may lead to the proteolysis of damaged proteins through targeting to the proteasome or may trigger autophagy and protein degradation by lysosomal enzymes [2]. The heat shock response is highly significant in human pathology, as HSP levels increase in cancer and promote tumorigenesis and decline in protein aggregation disorders such as Alzheimer’s disease, a lesion that permits accumulation of lethal protein inclusion bodies [3]–[6]. These effects seem to involve age-dependant deregulation of heat shock factor 1 (HSF1), the transcription factor that controls HSP expression. Further knowledge of the mechanisms of HSF1regulation during protein stress is thus highly desirable for developing an understanding the etiology of these disorders. We have examined the role of phosphorylation in regulating HSF1and showed that the factor is multiply phosphorylated on serine residues [7]. Some of these modifications are inhibitory for transcription, when HSF1 is phosphorylated on serines 121, 303, 307 or 363, or may be activating when HSF1 is phosphorylated on serine 320 by protein kinase A [8]–[12]. In this study, we have examined the role of the kinase mTOR in regulating the stress response and HSF1 phosphorylation and HSP mRNA and protein expression.

mTOR (*Mammalian target of rapamycin*) plays important roles in a responses to stress, including activation of the autophagy response in nutrient stress [13]. mTOR is a serine/threonine kinase distributed within two protein complexes in the cell [14]. One of these is the mTORC1 complex, containing mTOR and the adapter protein RAPTOR essential for phosphorylation of substrates [14]. mTORC1 is activated by signals from cell surface receptors that induce the kinase Akt [15]–[16]. Akt then phosphorylates the inhibitory factor TSC2, permitting mTORC1 activation [14]–[16]. mTORC1 is the target of rapamycin due to its interaction with/requirement for the rapamycin target molecule - immunophilin FKBP12 [14], [17], [18]. In addition, a second complex, mTORC2 has been discovered that is independent of upstream Akt signaling and in fact may function independently of the growth factors upstream of mTORC1 [14]. mTORC2 does not contain RAPTOR, but instead has a protein of homologous function, RICTOR [14]. mTORC1 plays an essential role in metabolism, strongly promoting mRNA translation and growth in cell size when nutrients are abundant and inhibiting macroautophagy under these conditions. Nutritional deprivation inhibits mTORC1 and promotes autophagy [15], [16]. It may be significant that HSF1 requires glutamine for activity (essential for mTORC1), promotes translation, one of the earliest findings in study of HSP expression [19]–[20]. HSF1−/− cells are significantly reduced in size, consistent with a role for HSF1 in cell size and perhaps mTORC1 function [21], [22].

## Materials and Methods

### Cells, Culture Condition and Reagents

HeLa cells were purchased from The American Type Culture Collection and cultured in DMEM (Invitrogen) supplemented with 10% heat inactivated fetal bovine serum (Invitrogen) and 1000 U of penicillin/streptomycin (Invitrogen) and maintained at 37°C in a 5% CO_2_ humidified incubator. Cells were treated with rapamycin (EMD Chemicals), KU0063794 (Selleck), Staurosporine (Selleck), 17-AAG (Sigma-Aldrich), MG132 (Sigma- Aldrich), Cadmium Chloride (Sigma-Aldrich) and Sodium Arsenite (Sigma-Aldrich) for 2 hours prior to treatment with heat at 43°C for 30 min.

**Figure 1:**
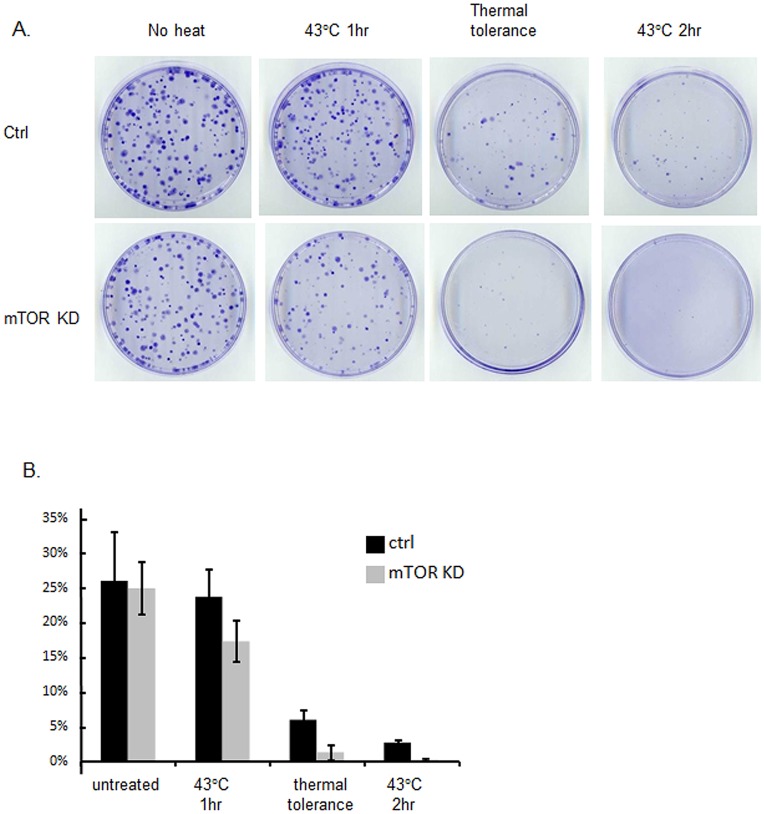
The role of mTOR in cellular resistance to proteotoxic stress. Control and mTOR knockdown HeLa cells were either untreated or treated with heat shock at 43°C for 1 hr and 2 hr. For thermal tolerance experiment, cells were treated with heat shock at 43°C for 1 hr and recovered at 37°C overnight. Cells were then treated with a second heat shock at 43°C for 2 hr followed by 37°C recovery. Nine days post-treatment, colonies were visualized by crystal violet staining. Colony counts were the averages calculated from three individual plates. Percentage of colonies formed was determined by comparison to the number of cells initially seeded in each treatment. Experiments were carried out three times, with reproducible findings.

### Plasmids and Transfection

All transfections were carried out with Fugene HD transfection reagent (Roche) according to manufacturer’s instructions. pGL3- Hsp70-LUC reporter construct, the GST-HSF1 expression plasmid (Murshid et. al., 2010) and the EGFP-HSF1 WT and EGFP-HSF1 S326A were both produced in the Calderwood lab and described in previous publications. Lentiviral short hairpin RNA (shRNA) expression vector for mTOR and the control pLKO.1 plasmid were purchased from Addgene and the myc-tagged mTOR expression plasmid was a kind gift from Dr. David Sabatini (Whitehead Institute).

### Lentiviral Production and Generation of mTOR Knockdown Cells

To generate the lentiviruses, envelope plasmid, packaging plasmid (Open Biosystems) and shRNA expressing plasmid were co-transfected into HEK293FT cells (Invitrogen). Virus-containing medium was collected 48 and 72 hr after transfection. HeLa and cells were infected by incubation with the lentivirus-containing medium and cells were treated with puromycin for selection of mTOR knockdown cells.

### Western Blot Analysis of Protein Expression

Cells were lysed with RIPA buffer containing protease inhibitor cocktail (Roche) and phosphatase inhibitor (Roche). Protein concentration was quantified with BCA protein assay kit (Pierce) and samples were subjected to SDS-PAGE followed by standard Western blot procedure. The following antibodies were used for analysis of protein expression: anti-Hsp70 (Enzo Life Sciences), anti-Hsp90 (Enzo Life Sciences), anti-Hsp110 (Enzo Life Sciences), anti-HSF1 (Enzo Life Sciences), HSF1 phospho-Serine 326 (Abcam), HSF1 phospho-Serine 303 (Abcam), HSF1 phospho-Serine 320 (Abcam), mTOR, p70 S6 Kinase (Cell Signaling), phospho-S6K Threonine 389 (Abcam), β-actin (Sigma-Aldrich) and GAPDH (Abcam). Secondary antibodies used were HRP-goat anti-at IgG, HRP-goat anti-mouse IgG, HRP-goat anti-rabbit IgG (Santa Cruz) and goat anti-rabbit IR Dye 680 (Licor).

**Figure 2:**
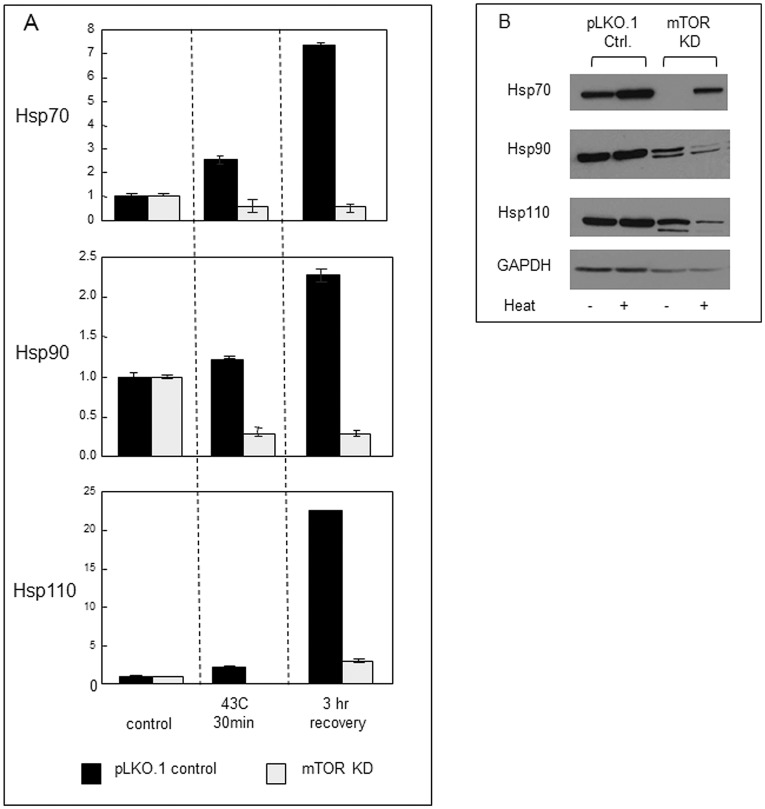
mTOR regulates transcriptional and translational expression of heat shock proteins. Control and mTOR knockdown HeLa cells were treated with or without heat. (A) Real time quantitative RT-PCR analysis was performed for analysis of the expression levels of Hsp70, Hsp90 and Hsp110 mRNA. Fold change was calculated by normalization to β-actin levels, followed by comparison with the control untreated sample. (B) Expression levels of Hsp70, Hsp90 and Hsp110 were then determined 24 hr after recovery from heat shock, at 37°C by western blotting using anti-Hsp70, anti-Hsp90 and anti-Hsp110 antibodies. Levels of GAPDH expression were also measured as loading controls. Experiments were carried out three times with consistent results.

### In Vitro Kinase Assay and Mass Spectrometric Analysis of Tryptic Peptides

Recombinant GST-HSF1 grown in *E. coli* was isolated and eluted with a glutathione column (Pierce) according to the manufacturer’s instructions. Recombinant GST-HSF1 was incubated with (100, 250 ng) or without recombinant mTOR (EMD Chemicals) in reaction mixture containing 10 µM ATP (Sigma-Aldrich), 2 mM DTT (Promega), 1× mTOR kinase buffer (Invitrogen) and protease inhibitor cocktail. Reaction mixture was incubated at 37°C for 30 min. To terminate the reaction, SDS sample buffer was added to the mixture and boiled at 95°C for 5 min. Reaction mixture was then subjected to SDS-PAGE and Western blot performed for detection of HSF1 Serine 326 phosphorylation. Reaction mixture resolved by SDS-PAGE was also stained by Coomassie blue to visualize GST-HSF1 and mTOR. The stained band corresponding to the GST-HSF1 was excised along with a blank band from another lane for Mass Spectrometric analysis by the Taplin Mass Spectrometry Facility (Harvard Medical School).

### Luciferase Assay

Transfection of the pGL3-Hsp70.1-LUC reporter construct was performed as described above. A β-galactosidase expression plasmid (pCMV-LacZ) was co-transfected with the reporter constructs as a control for transfection efficiency. Cell extracts were prepared in passive lysis buffer (Promega) and incubated on ice for 15 min followed by centrifugation. Protein concentration was determined by BCA protein assay. Both β-galactosidase and luciferase assays were performed according to standard procedures provided by the manufacturer (Promega). Luciferase activity was based on normalization to the β-galactosidase activity.

### RNA Harvesting, cDNA Preparation and Real-time Quantitative PCR

RNA was harvested with the RNeasy mini Kit (Qiagen) and reverse transfection carried out with a High capacity cDNA Reverse Transcription Kits (Applied Biosystems) following instructions provided by the manufacturers. For analysis of HSP expression, real-time quantitative PCR was performed using FastStart Universal SYBR green (ROX) master mix and primer pairs specific for the amplification of target genes. All reaction was performed on ABI 7300 Real-time PCR System. Thermocycling condition includes a 15 min hot start at 95°C followed by 40 cycles of 15 sec denaturation at 95°C and 1 min of annealing and extension at 60°C. Hsp70B primers (forward 5′-ACCTTCCCCGCATTTCTTTCAGCA, reverse 5′-CCGCGGTAGCATACGCGCA); Hsp90 primers (forward 5′-TGGTAGACACAGGCATTGGCATGA, reverse 5′-AGCCAACACCAAACTGCCCAATCA); Hsp110 primers (forward 5′-ACTGCTTGTTCAAGAGGGCTGTGA, reverse 5′-AACATCCACACCCACACACATGCT). Sample for each treatment group were performed in triplicates and experiments repeated three times. Data analysis was done by using the comparative C_t_ method with β-actin [9] as normalization control.

**Figure 3:**
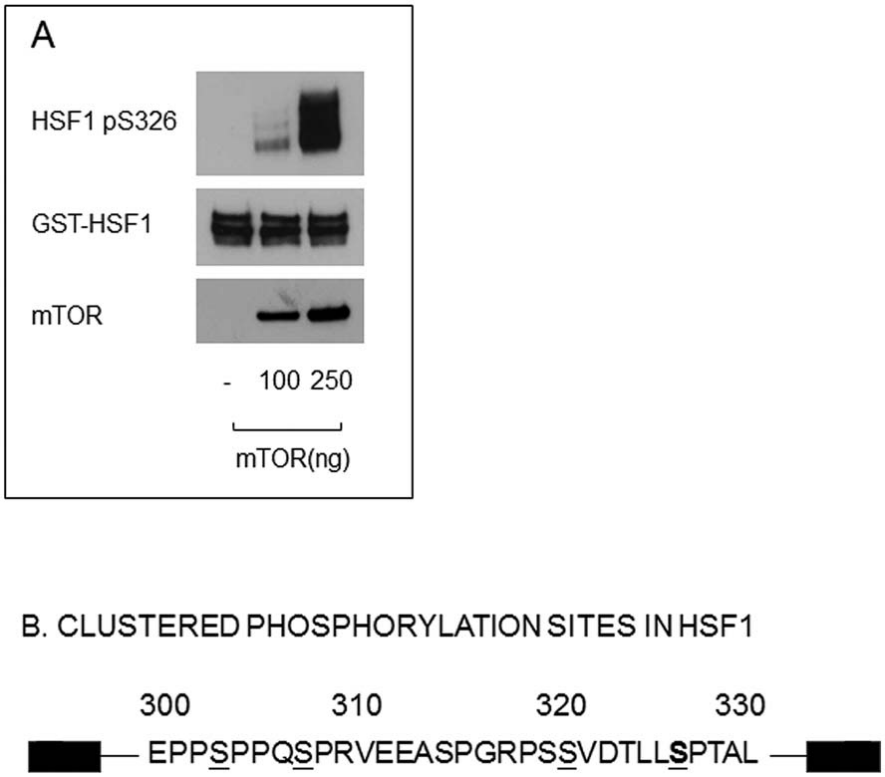
Purified GST-tagged HSF1 was phosphorylated *in vitro* by mTOR. (A) *In vitro* phosphorylation was performed by mixing GST-tagged HSF1 with various concentrations of recombinant mTOR. Samples were then fractionated by 10% SDS-PAGE and western analysis performed to examine the levels of HSF1-phosphoserine 326 and total HSF1. The identities of the phosphopeptides were determined by isolating the mTOR phosphorylated GST-HSF1 with 10% SDS-PAGE, trypsin digestion of the GST-HSF1 band and identification of peptides by mass spectrometry and database analysis. (B) Protein sequence of HSF1 from amino acids 300–330. The mTOR phosphorylation site, **serine 326** is marked in bold and nearby serine phosphorylation sites that have been characterized (serines 303, 307, 320 and 326) are underlined.

### Detection of Cell Viability by Crystal Violet

Cells (10^3^ cells/plate) suspended in DMEM medium with 10% FBS were added to 6 cm flat-bottomed plate. Cells were treated with or without heat and incubated at 37°C for nine days for recovery. The supernatants were discarded and the remaining viable adherent cells were fixed with methanol for 10 min then stained with 0.5% crystal violet in 25% methanol for 10 min. Cells were then rinsed with water and allow to dry overnight.

## Results

### Role of mTOR in Cellular Resistance to Proteotoxic Stress

We first investigated the role of mTOR in cellular responses to proteotoxic stress after heat shock. Activation of the heat shock response leads to the synthesis of HSPs that regulate resistance to an array of stresses, including heat itself [1]. We examined the ability of mTOR to regulate the sensitivity to heat shock of HeLa cells, as assayed by clonogenic cell survival (Fig. 1). The HeLa cell strain used in these studies was approximately 25% clonogenic prior to stress. Exposure to heat shock for 1 hr at 43°C led to mild levels of cell inactivation as indicated by a reduction in colony formation and killing was increased by decreasing mTOR levels with shRNA treatment (Fig. 1). Exposure to 2 hr at 43°C led to pronounced cell inactivation and survival was further decreased by mTOR knockdown. However, pretreatment of cells with a priming heat shock (1 hr at 43°C) made them resistant to cell killing by a second heat shock 2 hr at 43°C (Fig. 1). Such stress resistance was antagonized by mTOR knockdown using shRNA and a marked reduction in colony formation was observed.

**Figure 4:**
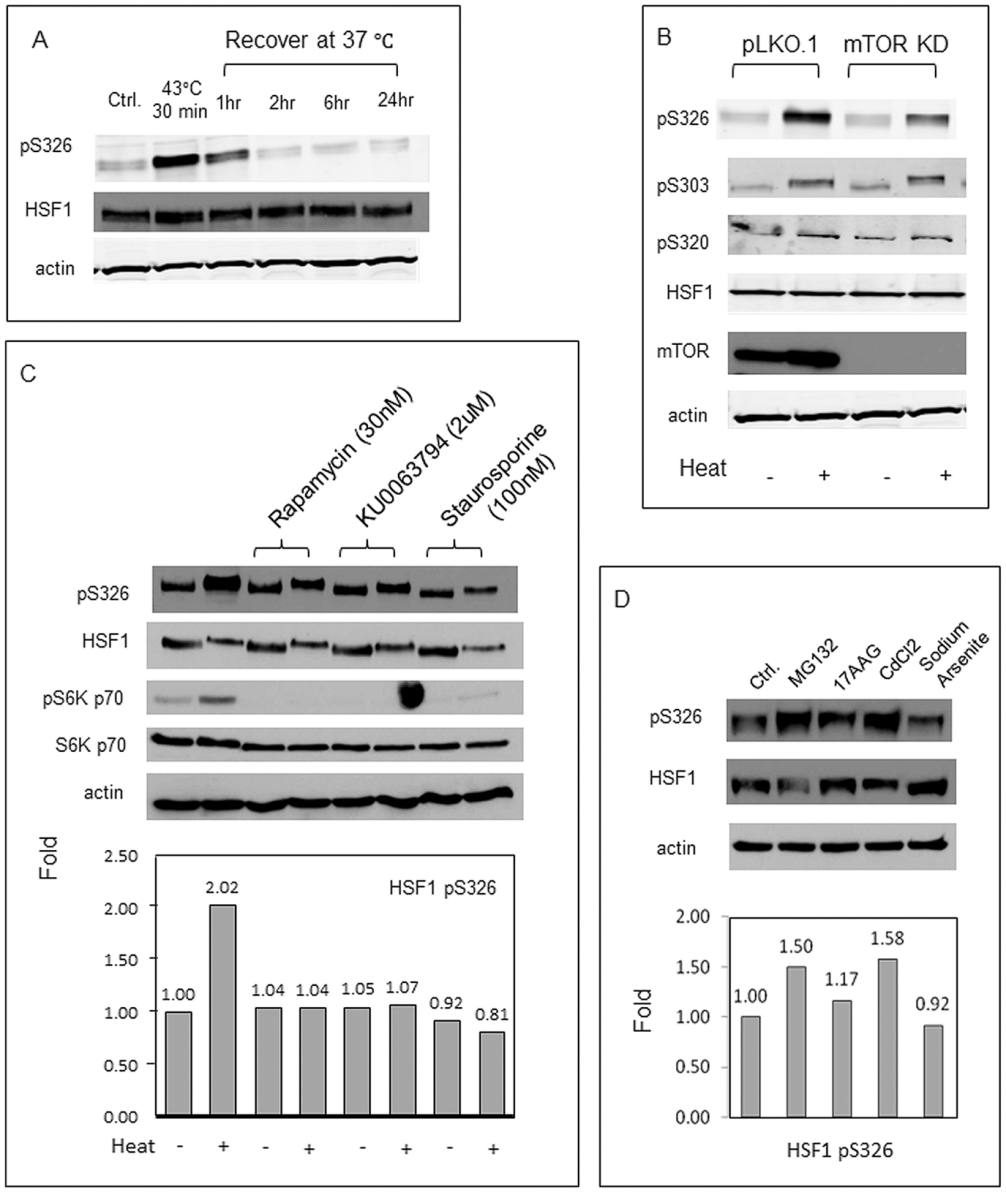
Regulation of HSF1 serine 326 phosphorylation during stress. (A) Levels of HSF1-phosphoserine 326, total HSF1 and β-actin in HeLa cells treated with heat at 43°C for 30 min and recovered at 37°C for up to 24hr. (B) Levels of HSF1-phosphoserine 326, HSF1-phosphoserine 303, HSF1-phosphoserine 320, total HSF1, mTOR and β-actin in control and mTOR knockdown HeLa cells with or without heat shock at 43°C for 30 min. (C) Intracellular concentration of HSF1-phosphoserine 326, total HSF1, S6 kinase-phosphothreonine-389, total S6 kinase and β-actin, without or with heat shock in HeLa cells pretreated with mTOR inhibitors rapamycin (30 nM) and KU0063794 (2 µM) and kinase inhibitor staurosporine (100 nM) for 2 hr. Relative levels of HSF1-phosphoserine 326 in cells after the various treatments were determined by densitometric analysis of X-ray films, normalized to untreated cells (lane 1), and are indicated below the representation of the immunoblots. (D) HeLa cells were treated for 2 hr with stress inducers MG132 (5 µM), 17-AAG (2 µM), CdCl_2_ (200 µM) and sodium arsenite (1 µM) prior to assay for HSF1-phosphoserine 326, total HSF1 and β-actin. Relative levels of HSF1-phosphoserine 326 in cells were determined by densitometric analysis as above, normalized to levels in untreated cells (lane 1), and are indicated below the representation of the immunoblots. Experiments were performed on at least three occasions with reproducible findings.

### Effects of mTOR Silencing on HSP Synthesis

As temperature sensitivity has been shown to be tightly regulated by HSP expression, we therefore next examined the effects of mTOR knockdown on expression of three key HSPs- Hsp70, Hsp90 and Hsp110 (Fig. 2) [23]. Heat shock led to the abundant expression of Hsp70, Hsp90 and Hsp110 mRNA species as determined by realtime quantitative PCR (Fig. 2A). mTOR knockdown, abolished the induction of each mRNA species while not affecting basal expression of the HSP mRNAs in control cells maintained at 37°C (Fig. 2A). In addition, mTOR knockdown decreased the expression of each of the proteins as indicated by immunoblot assay (Fig. 2B). Part of this decrease in protein synthesis may however be attributed to a decline in general translation that accompanies reduced mTOR levels and levels of housekeeping proteins such as GAPDH (Fig2B) and β-actin (not shown) are also decreased when mTOR levels are reduced.

### HSF1 as a Substrate for the Phosphotransferase Activity of mTOR

Expression of HSPs and enhanced heat resistance are regulated by the transcriptional activity of HSF1 in heat shocked cells and we therefore examined whether this factor is a potential target for mTOR in the stress response. We first examined the ability of mTOR to phosphorylate HSF1 *in vitro*. Inspection of the HSF1 sequence for potential mTOR phosphorylation sites indicated the amino acid motif surrounding serine 326 bears a sequence resemblance to AGC kinase substrates of mTOR [17], [24]. Although a consensus phosphorylation motif has not been derived for mTOR, this kinase frequently phosphorylates threonine or serine residues flanked by a proline residue in the +1 position and a hydrophobic residue at −1. The S326 residue has proline at +1 and the highly hydrophobic leucine at −1 (Fig. 3). Indeed, incubating purified GST-HSF1 *in vitro* with increasing concentrations of purified mTOR and ATP led to kinase dose-dependent phosphorylation of HSF1 on serine 326 as determined by an antibody specific for HSF1-phospho S326 (Fig. 3 A). Proteins incubated with recombinant mTOR were then isolated, subjected to protease digestion and analyzed by mass spectrometry using an LTQ-Orbitrap (Thermo Electron). Two main peptide species contained phosphorylated residues after exposure to mTOR and these were: VEEASPGRPSSVDTLLS#PTALIDSILR (V25R), containing S326 and VKEEPPSPPQSPR (V11R) containing the previously characterized phosphoserine 303 [7]. However as subsequent studies shown later in the manuscript indicate that HSF1-S303 phosphorylation is not altered *in vivo* by inhibition of mTOR activity, we pursued a role for mTOR in regulating the stress response through phosphorylation of S326 in HSF1. A previous phosphorylation screen of HSF1 suggested the importance of this residue in transcriptional regulation during stress [25].

**Figure 5:**
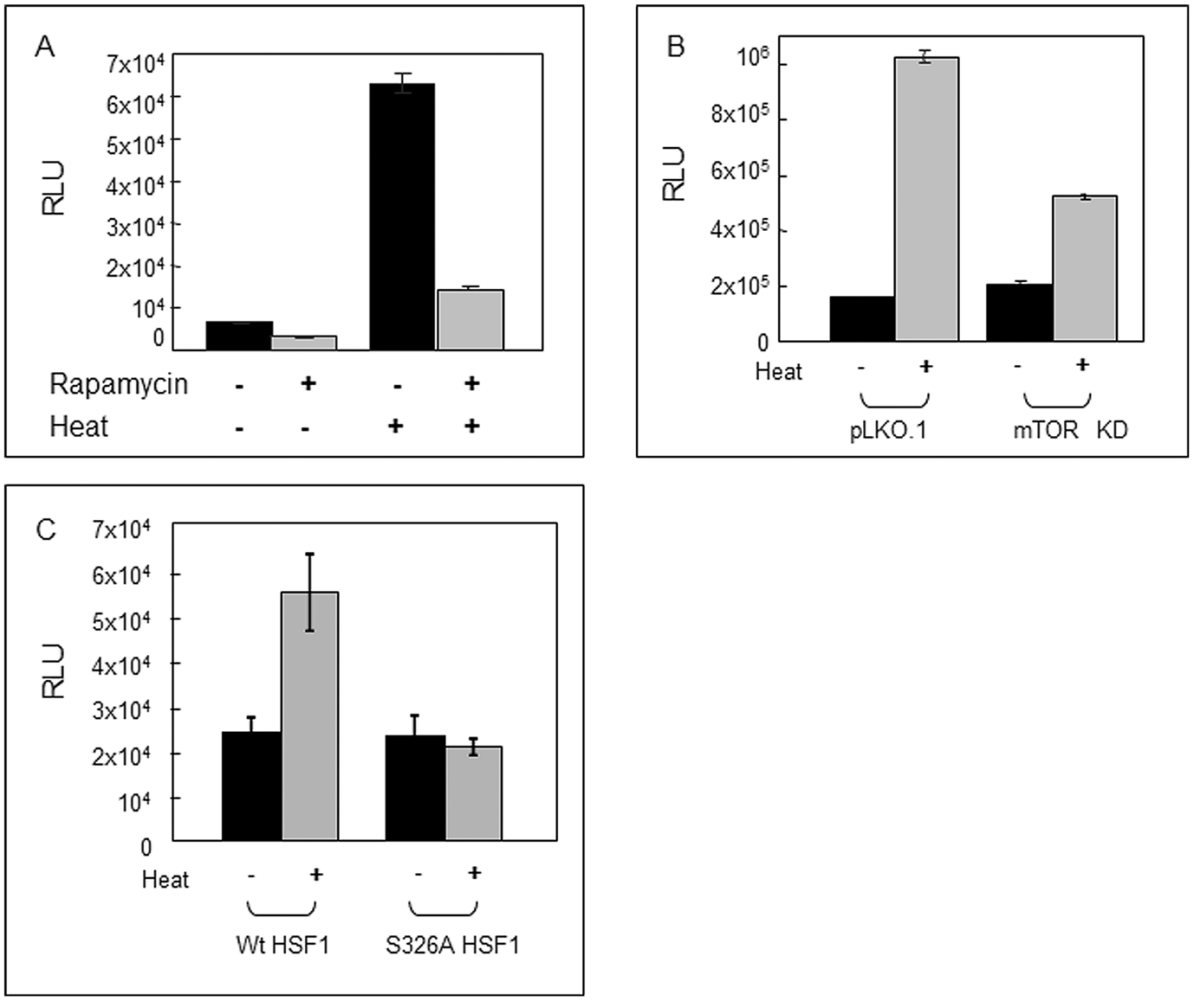
mTOR activity and serine 326 phosphorylation are required for the heat induced activation of the *hsp70.1* promoter. (A) Effects of 43°C heat shock on *hsp70.1* activity in HeLa cells without and with rapamycin treatment. Cells transfected with the pGL3-Hsp70-LUC reporter construct were treated with 30 nM rapamycin for 2 hr prior to receiving heat shock and luciferase activity was assayed at 24 hr. Data are means of triplicate assays +/− SD. (B) Effects of mTOR knockdown on activation of the *hsp70.1* promoter by heat shock. HeLa cells either stably expressing shRNA targeted to mTOR or control hairpin were transfected with the pGL3-Hsp70-LUC reporter construct followed by heat shock (effectiveness of mTOR knockdown is indicated in Fig. 4B). Luciferase activity in cells after 48 hr recovery at 37°C is the mean of triplicate assay and is plotted +/− SD (C) HeLa cells depleted of HSF1 by stable expression of shRNA targeting the factor were co-transfected with the pGL3-Hsp70-LUC reporter construct and expression plasmids encoding either wild type HSF1 or HSF1-S326A. Cells lysates were collected from cells that were either heat shocked or used as control, 48 hr post-heating and luciferase assays were then performed in triplicate and activity plotted as mean +/− standard deviation. Experiments were carried out in duplicate, reproducibly.

### Regulation of HSF1 Phosphorylation on Serine 326 by Heat Shock

We next investigated HSF1-S326 phosphorylation *in vivo* in HeLa cells subjected to stress (Fig. 4A). Cells were exposed to heat shock at 43°C for 30 min and then assayed for HSF1-S326 phosphorylation using the antibodies specific for this phosphorylated residue. Phosphorylation on S326 was detected immediately after heat shock and persisted for approximately 1 hr of recovery at 37°C, the approximate period of HSP gene transcription. While the overall levels of HSF1 protein were unaffected, HSF1 phosphorylation after stress *in vivo* appeared to be dependent on mTOR activity as knockdown of the kinase by shRNA led to the inhibition of stress-induced HSF1-S326 phosphorylation, while exposure to control oligonucleotides did not affect activity (Fig. 4B). This effect of stress appears to have some specificity for S326 phosphorylation as phosphorylation of HSF1 at two adjacent sites- S303 and S320, that can each regulate HSF1 activity - was not affected significantly by mTOR knockdown (Fig. 4B) [8], [9]. We also detected basal levels of HSF1-S326 phosphorylation in unstressed HeLa cells that were not affected by mTOR knockdown suggesting that this site may be a target for other protein kinases in non-heat shock conditions. We next examined the effect on HSF1-S326 phosphorylation of drugs that have been shown to inhibit mTOR activity, including rapamycin, KUOO6379 and staurosporine. Heat shock led to phosphorylation of HSF1 on S326 (Fig. 4C). Each of the drugs inhibited the stress-induced activation of HSF1-S326 phosphorylation while abolishing the phosphorylation on T389 of ribosomal S6 kinase, a well-characterized substrate for mTOR (Fig. 4C). Inhibition of HSF1 phosphorylation on S326 by rapamycin suggests that this site in HSF1 is a target for the TORC1 complex (Fig. 4C). Other stresses including exposure to proteasome inhibitor MG132 or sodium arsenite increased the levels of HSF1-S326, although some other HSP inducers such as heavy metal ion Cd^++^ or the Hsp90 inhibitor 17-AAG did not significantly activate phosphorylation (Fig. 4D). Heat shock appeared to be the most potent activator of HSF1-S326 phosphorylation (Fig. 4C). We also investigated whether HSF1 could bind stably to mTOR, as HSF1 has been shown to associate with a number of protein kinases [9], [26]. However we failed to observe mTOR binding to HSF1 in immunoprecipitation with the anti-HSF1 antibodies, suggesting a transient interaction of HSF1 with mTOR (S-D Chou & SK Calderwood, data not shown).

### HSF1 Phosphorylation by Heat Shock Regulates the HSP70 Promoter

HSF1 is a powerful inducer of HSP promoters after stress. We therefore examined the effects of mTOR inhibition on the promoter of the heat shock-inducible *hsp70.1* gene using an *hsp70.1* promoter-luciferase reporter construct (Fig. 5). Heat shock strongly activated the *hsp70.1* promoter and this activity was inhibited by exposure to rapamycin, indicating a role for the TORC1 complex in activating the transcriptional activity of HSF1 (Fig. 5A). In addition, knockdown of mTOR with shRNA also reduced *hsp70.1* promoter activation by heat shock (Fig. 5B). A role for TORC1 in these effects is also indicated by the fact that stress-induced *hsp70.1* activity is inhibited by shRNA targeted to TORC1 complex component *raptor* but not by similar inhibition of *rictor*, a protein essential for the TORC2 complex (unpublished data). Finally we investigated the role of modification of HSF-S326 by alanine substitution on ability to activate transcription of the hsp70.1 promoter (Fig. 5C). In the HSF1 knockdown HeLa cells, ectopic expression of the wild type HSF1 allows the activation of the *hsp70.1 promoter* in response to heat as expected, however, expression of the mutant S326A HSF1 failed to activate the heat shock response.

## Discussion

Although some of the features of heat shock factor regulation have been known since the early days of study of transcription, a complete picture has been slow in coming [26]. It was long suspected that complete HSF1 activation requires stress-induced posttranslational modifications including phosphorylation [26]–[28]. Our current studies indicate that the TORC1 complex may play a critical role in HSF1 activation at least partially through direct phosphorylation of HSF1 on a residue required for transcription- serine 326. We show that HSF1 activation may be dependent on TORC1 activation during stress (Fig. 3). The mechanism whereby TORC1 triggers the *trans*-activating activity of HSF1 is not revealed here. However, phosphorylation of AGC kinases by mTOR on a consensus site that resembles the motif surrounding S326 leads to their independence from Hsp90 chaperoning [24]. HSF1 is known to be released from Hsp90 chaperone complexes during activation by heat shock, and this mechanism may require phosphorylation by mTOR [29]. We have recently shown that a serine residue closely adjacent to the mTOR target site -serine 320 is also important in HSF1 activation by proteotoxic stresses and when phosphorylated promotes nuclear localization of HSF1 and recruits an activating complex containing the histone acetylase p300 as well as pTEF-b to HSP promoters. This mechanism appears to be largely independent of mTOR and S326 phosphorylation, as mTOR inhibition does not affect the levels of S320 phosphorylation (Fig. 4B) and did not alter stress-induced nuclear localization of the HSF1 after heat shock (data not shown). Furthermore S320 phosphorylation depends on the activity of protein kinase A [9], [10]. The region of HSF1 from residue 300 to 330 contains at least six residues shown to be phosphorylated *in vivo* (S303, S307, S314, S320, T323 and S326; Fig. 3B) [7], [9], [10], [12], [30], [31]. Phospho-S303 and phospho-S307 each inhibit HSP gene transcription, phospho-S320 and phospho-S326 activate transcription while the potential roles of phospho-S314 and phospho-T323 are unknown. HSF1 activation in stress appears to be a multi-step process, involving a series of phosphorylation events, largely clustered in the 300–330 amino acid region, which lead to rapid and efficient regulation of stress genes and guardianship of the proteome (Fig. 3B).

As mTOR is a highly pleiotropic kinase it is possible that it plays other roles in the proteotoxic stress response. For instance, TORC1 activation by mitogens amplifies mRNA translation by a complex pathway involving downstream activation of ribosomal S6 kinase [32]. However, heat shock appears to have the opposite effect on translation and strongly inhibits protein synthesis as it can inactivate S6 kinase [33] (data not shown). Inhibition of translation during heating appears instead to involve other effects on cell signaling such as the activation of eIF2 alpha kinases by phosphorylation. Phosphorylated eIF2 alpha is a dominant inhibitor of translation and results in sequestration of mRNAs into cytoplasmic stress granules [34]. In addition, activation of TORC1 has been shown to inhibit the autophagy pathway [35]. This is again counter-intuitive in light of our findings, as most studies indicate activation of autophagy by heat shock [36]. However, our unpublished observations indicate that transcriptional activation of autophagy during stress is a complex process involving mechanisms independent of TORC1 signaling (Y Zhang & SK Calderwood, in preparation).
